# JAK inhibitors in rheumatoid arthritis-associated interstitial lung disease: a review of mechanisms and clinical evidence

**DOI:** 10.3389/fimmu.2026.1784714

**Published:** 2026-07-01

**Authors:** Zhiying Wu, Li Jiang, Xu Zheng, Wanlin Zhou, Zuofeng Wang, Weilin Xie

**Affiliations:** 1 Military Treatment Center, The 970th Hospital of the People’s Liberation Army Joint Logistics Support Force, Yantai, China; 2 The Department of Oncology, The 970th Hospital of the People’s Liberation Army Joint Logistics Support Force, Yantai, China; 3 The Department of Traumatic Orthopedics, The 970th Hospital of the People’s Liberation Army Joint Logistics Support Force, Yantai, China

**Keywords:** JAKi, rheumatoid arthritis, interstitial lung disease, mechanisms, clinical evidence, treatment, review

## Abstract

Janus kinase (JAK) inhibitors, as oral targeted synthetic disease-modifying anti-rheumatic drugs (DMARDs), represent a promising therapeutic option for rheumatoid arthritis-associated interstitial lung disease (RA-ILD). Preclinical studies suggest a dual mechanism, inhibiting the pro-fibrotic interleukin-17A (IL-17A)/JAK2/nuclear factor kappa B (NF-κB) axis in lung fibroblasts and modulating lung immunity. Clinical evidence, primarily from observational studies and meta-analyses, indicates that JAK inhibitors do not increase the risk of incident ILD and may stabilize or improve lung function and radiological findings in established RA-ILD, showing comparable efficacy to abatacept or rituximab. However, recent real-world data highlight potential safety concerns, suggesting a higher risk of mortality and severe outcomes compared to tumor necrosis factor (TNF) inhibitors, particularly in older patients or those with cardiovascular comorbidities. While effective, the use of JAK inhibitors in RA-ILD requires careful patient selection and risk-benefit assessment, underscoring the need for confirmatory randomized controlled trials.

## Introduction

Rheumatoid arthritis-associated interstitial lung disease (RA-ILD) is the most common and serious extra-articular manifestation of RA. A recent systematic review and meta-analysis informed by data from 69 studies encompassing 7879 patients confirms its severity and association with significantly increased morbidity and mortality rates compared to RA without pulmonary involvement, imposing substantial healthcare costs (1). Its management is challenging, requiring simultaneous control of joint inflammation, halting pulmonary progression, and avoiding drug-induced lung injury. For the rheumatologist, this represents a complex balancing act between systemic immunosuppression and pulmonary safety.

The development of RA-ILD is influenced by a combination of genetic, environmental, and immunological factors. Established risk factors include older age, male sex, smoking history, high titers of rheumatoid factor (RF) and anti-cyclic citrullinated peptide (anti-CCP) antibodies, and more severe articular disease (2). A recent systematic review by Akiyama et al. (2022) further highlights the role of specific autoantibodies and genetic polymorphisms in driving pulmonary involvement, underscoring the multifactorial nature of RA-ILD pathogenesis (3). These risk factors not only predispose patients to ILD but may also influence disease progression and response to therapy, thereby informing clinical surveillance and treatment strategies.

In recent years, non-conventional synthetic disease-modifying anti-rheumatic drugs (ncs-DMARDs), including biologic DMARDs (bDMARDs) and targeted synthetic DMARDs (tsDMARDs), have provided new options. A 2023 systematic review and meta-analysis indicated that ncs-DMARDs could stabilize lung function and high-resolution CT (HRCT) findings in most RA-ILD patients and reduce ILD-related mortality (4). Among these, janus kinase (JAK) inhibitors, as oral tsDMARDs, have garnered significant interest due to their broad immunomodulatory effects. A 2024 systematic review and meta-analysis by Narváez et al., which included 183 patients from observational studies, provides a comprehensive synthesis of the efficacy and safety of JAK inhibitors in RA-ILD (5).

The pathogenesis of RA-ILD involves excessive formation of tertiary lymphoid organs (inducible bronchus-associated lymphoid tissue, iBALT) in the lungs, local autoantibody production, and subsequent neutrophil activation leading to fibrosis (3). This understanding underscores the rationale for immunomodulatory therapies. However, a recent large propensity score-matched study found no significant differences in mortality or respiratory hospitalization between RA-ILD patients initiating rituximab, abatacept, tocilizumab, or tofacitinib, though estimates were imprecise (6). This highlights the ongoing uncertainty in comparative effectiveness and the need for more evidence, particularly regarding JAK inhibitors. Current studies focusing on JAK inhibitors in RA-ILD remain scarce, with evidence primarily derived from observational studies and case series (5, 7, 8), which inherently limits the strength of recommendations.

## Mechanistic rationale: preclinical insights into the IL-17A/JAK2/NF-κB axis and immunomodulatory effects

JAK inhibitors exert their effects by blocking the JAK-signal transducer and activator of transcription (STAT) signaling pathway, central to the transduction of signals for numerous cytokines implicated in both inflammation and fibrosis. Preclinical research provides a strong mechanistic foundation. Of particular relevance is the direct role of the interleukin (IL)-17A/IL-17RA axis in human lung fibroblast activation. Zhang et al. demonstrated that both normal and fibrotic human lung fibroblasts express functional IL-17RA and respond to IL-17A stimulation with proliferation, extracellular matrix (ECM) production, and myofibroblast trans-differentiation (9). This pro-fibrotic response was dependent on nuclear factor kappa B (NF-κB) signaling. Critically, they showed that siRNA silencing of JAK2, or its inhibition with the selective inhibitor AZD1480, significantly attenuated this IL-17A-induced fibrogenic response, including downstream NF-κB activation and IL-17RA expression. In contrast, inhibition of JAK1/JAK3 with tofacitinib suppressed STAT3 phosphorylation but did not affect the fibrogenic output, indicating a specific and essential role for JAK2 in this pathway (9). This preclinical evidence directly links the IL-17 pathway, known to be active in RA, to a JAK2/NF-κB-driven fibrotic mechanism in lung fibroblasts. This suggests that JAK inhibitors with significant JAK2 inhibitory activity (e.g., baricitinib, ruxolitinib) might theoretically offer a more direct anti-fibrotic effect in the lung, although this requires clinical confirmation.

Further supporting the immunomodulatory role of JAK inhibitors, a study in the SKG mouse model of RA-ILD demonstrated that tofacitinib ameliorated interstitial lung disease by facilitating the expansion of myeloid-derived suppressor cells (MDSCs) in the lungs. These expanded MDSCs were shown to suppress cluster of differentiation (CD)4^+^ T cell proliferation and T help (Th) 17 cell differentiation ex vivo, providing a potential mechanism by which JAK inhibitors can modulate the local immune environment in the lung toward a less inflammatory and fibrotic state (10). This aligns with the known capacity of JAK inhibitors to concurrently modulate both inflammatory and fibrotic pathways, a dual action highly relevant for a disease like RA-ILD that encompasses both processes.

This is corroborated by other studies. Lescoat et al. (2020) demonstrated that JAK inhibitors, particularly those with activity against JAK2 such as ruxolitinib, and to a lesser extent tofacitinib (which primarily inhibits JAK1 and JAK3), can downregulate the expression of both pro-inflammatory macrophage (M)1 markers and pro-fibrotic M2 markers in human monocyte-derived macrophages *in vitro* (11). In an animal model of scleroderma-associated ILD, ruxolitinib treatment significantly reduced key M1 and M2 macrophage marker expression in the lungs, improved histopathological outcomes, and decreased hydroxyproline content, indicating attenuated fibrosis (11). The JAK/STAT pathway mediates the biological effects of key cytokines (e.g., IL-4, IL-6, IL-13, tumor necrosis factor (TNF)-α, interferon (IFN-γ) and growth factors (e.g., transforming growth factor (TGF)-β) pivotal for lung homeostasis (12). Different JAK inhibitors have varying selectivity: upadacitinib and filgotinib are JAK1-selective, while tofacitinib inhibits JAK1 and JAK3 > JAK2 and TYK2, and baricitinib targets JAK1 and JAK2 (12). This pharmacologic diversity implies that not all JAK inhibitors may have identical effects on RA-ILD, a nuance often lost in class-level discussions.

## Clinical evidence: JAKi efficacy in RA-ILD from recent meta-analyses and systematic reviews

A recent comprehensive systematic review and meta-analysis specifically evaluating treatments for RA-ILD, including 69 studies up to April 2025, provides valuable context for JAK inhibitor efficacy (1). The analysis found that treatments including JAK inhibitors (JAKi) were associated with stabilization or improvement of forced vital capacity (FVC). Notably, in a meta-analysis of between-group differences in FVC% change (treated vs. non-users), most treatments, including JAKi, demonstrated beneficial effects with positive differences compared to controls, suggesting a treatment effect beyond the natural disease course. High heterogeneity was noted, but the consistency of direction across treatments strengthens reliability (1). Furthermore, this review highlighted that methotrexate (MTX) and abatacept (ABA) were associated with a reduced risk of disease progression and mortality, while tumor necrosis factor inhibitors (TNFi) showed a trend toward increased progression risk (1). These comparative data are important when positioning JAK inhibitors within the treatment landscape. For clinicians, this reinforces avoiding TNFi in patients with established RA-ILD and supports considering JAKi alongside abatacept and rituximab as preferred advanced therapies. To provide a structured overview of the clinical evidence, **Table 1** summarizes key studies evaluating JAK inhibitors in patients with RA-ILD.

**Table 1:** Summary of key clinical studies evaluating JAK inhibitors in RA-ILD.

Study (year)	Design	Sample size (RA-ILD)	JAK inhibitor(s)	Follow-up duration	Primary lung outcome	Safety notes
Narváez et al. (2024) (5)	Systematic Review & Meta-analysis	183	Various JAKi	Variable across studies	pFVC% (+2.07%), ↑pDLCO% (+3.12%), HRCT improvement (11%)	Pooled AE rate: 14%
Wang et al. (2023) (13)	Prospective Observational Cohort	24 (TOF+IGU arm)	Tofacitinib + Iguratimod	≥6 months	FVC% (90.7 ± 15.4 vs. 75.9 ± 6.8, P = 0.031), improved HRCT fibrosis score	Well-tolerated, no serious infections reported
TREASURE Registry (2022) (17)	Real-world Observational	27	Tofacitinib	Median 12 months	FVC% (+3.0%, p=0.014), worsening in 11.1%	Increased risk of respiratory infections noted
MAJIK-SFR Cohort (2025) (18)	Prospective Cohort	42	Various JAKi	Median 21 months	FVC/DLCO stable; HRCT stable (69%), improved (12%), worsened (19%)	Respiratory tract infections (38%, mostly mild)
Venerito et al. (2023) (8)	Multicenter Retrospective	43	Various JAKi	Median 19.1 months	HRCT stable (86%), FVC stable (78.6%)	No MACE, VTE, or lung cancer reported
Tsujii et al. (2024) (19)	Retrospective Nested Case-Control	21 (JAKi group)	Tofacitinib/Baricitinib	24 months	Improved HRCT ground-glass score (p=0.03)	Similar safety profile to abatacept

pFVC%, percent predicted forced vital capacity; pDLCO%, percent predicted diffusing capacity for carbon monoxide; HRCT, high-resolution computed tomography; AE, adverse events; MACE, major adverse cardiovascular events; VTE, venous thromboembolism.

All studies included adult patients with confirmed RA-ILD; safety outcomes are as reported in the respective publications.

## Clinical evidence: JAK inhibitor combination therapy in RA-UIP

Recent prospective data have explored the efficacy of JAK inhibitors in combination with other agents. A 2023 prospective observational cohort study by Wang et al. evaluated tofacitinib (TOF) combined with iguratimod (IGU) in RA patients with usual interstitial pneumonia (UIP) pattern ILD (RA-UIP) (13). The study compared TOF plus IGU (n=24), IGU plus csDMARDs (n=26), and csDMARDs alone (n=28) over at least 6 months. While all groups showed improvement in RA disease activity (DAS28-CRP, ESR, CRP), the TOF plus IGU group demonstrated a significant improvement in percent predicted forced vital capacity (FVC%) compared to the csDMARDs group (90.7 ± 15.4 vs. 75.9 ± 6.8, P = 0.031). HRCT fibrosis scores also improved significantly in the TOF+IGU group versus the csDMARDs group (7.0 ± 5.6 vs. 7.9 ± 5.2, P = 0.015). Furthermore, the TOF plus IGU group had a significantly higher proportion of patients with HRCT regression or stability (66.7%) compared to the csDMARDs group (35.7%, P = 0.027) (13). The authors proposed that this combination, leveraging both anti-inflammatory (JAK inhibition) and potential anti-fibrotic (IGU) properties, may represent a “dual treat-to-target” strategy for simultaneously addressing joint and lung disease (13). However, this study was limited by its small sample size, single-center design, and short follow-up period. The concept is intriguing, but such combinations should be considered experimental until validated in larger, controlled studies.

## Clinical evidence: impact on the risk of developing ILD (incident ILD)

A critical question is whether DMARDs increase the risk of new-onset (incident) ILD. A 2024 systematic review and meta-analysis by Zhang et al., encompassing 40 studies and over 486,000 RA patients, provides key insights (14).

Furthermore, a large retrospective cohort study by Baker et al. (2023) specifically investigated the incidence of ILD in RA patients initiating different b/tsDMARDs. Among 28,559 patients, the crude incidence rate of ILD per 1000 person-years was lowest for tofacitinib (1.47; 95% CI 0.54–3.27), compared to adalimumab (3.43), abatacept (4.46), rituximab (6.15), and tocilizumab (5.05). After multivariable adjustment, tofacitinib was associated with a 69% reduced risk of developing ILD compared to adalimumab (adjusted HR 0.31; 95% CI 0.12–0.78). This protective association remained robust in a sensitivity analysis using a prevalent new-user design (adjusted HR 0.32; 95% CI 0.13–0.82) (15).

Tofacitinib: Pooled analysis of randomized controlled trials (RCTs), including the large ORAL Surveillance trial, found no significant difference in the odds of ILD between tofacitinib and TNF inhibitors (OR 0.94, 95% CI 0.52–1.69) (14). However, a large retrospective cohort study within the same analysis reported a significantly lower odds of incident ILD with tofacitinib compared to adalimumab (OR 0.36, 95% CI 0.15–0.87), a discrepancy the authors attribute to potential channeling bias in observational studies (14).

Overall Conclusion: The meta-analysis concluded that no DMARD, including JAK inhibitors, was associated with an increased risk of ILD in RCTs (14). This is supported by a pooled analysis from Narváez et al., which found a low incidence rate of *de novo* ILD in patients receiving JAK inhibitors (0.20 per 1000 person-years) (5). Furthermore, a large US cohort study found that tofacitinib was associated with a 69% reduced risk of ILD compared to adalimumab (5). Similar data for baricitinib also document a low risk of developing ILD during treatment (12). Notably, no study has proven an increased risk of ILD under JAK inhibitor treatment in RA (12). From a clinical standpoint, this reassuring safety data regarding incident ILD removes a major theoretical barrier to using JAK inhibitors in RA patients with risk factors for, but without established, ILD.

## Clinical evidence: efficacy in established RA-ILD

Regarding their role in treating established RA-ILD, evidence is emerging primarily from real-world studies and subgroup analyses. A recent propensity score-matched study comparing non-TNFi b/tsDMARDs found no significant difference in the primary composite outcome of death or respiratory hospitalization between tofacitinib and rituximab initiators over three years (adjusted HR: 0.89 [0.54, 1.46]) (6). This suggests comparable effectiveness on “hard” clinical outcomes between JAK inhibitors and a commonly recommended therapy like rituximab. Globally, observational studies report overall stabilization of respiratory symptoms, functional data, and radiographic extension of ILD (12).

Supporting Case Evidence: A 2023 case report described a 69-year-old man with drug-refractory RA-ILD (NSIP pattern) who failed multiple therapies including prednisolone, methotrexate, tacrolimus, abatacept, and intravenous cyclophosphamide, meeting criteria for progressive pulmonary fibrosis. After initiation of upadacitinib 15 mg daily, his dyspnea (mMRC grade), ground-glass opacities on HRCT, and pulmonary function (DLCO) improved within 29 weeks, allowing for dose reduction of both upadacitinib and prednisolone. This case highlights the potential of JAK inhibitors as a rescue therapy in refractory RA-ILD (16).

Findings from a 2023 Systematic Review: The meta-analysis by Yuan et al. showed that while overall forced vital capacity (FVC) did not change significantly after ncs-DMARD treatment, subgroup analysis revealed that rituximab significantly improved FVC (4). Data on JAK inhibitors from included studies suggested a trend toward slight improvement in DLCO (4). Crucially, the pooled results demonstrated that 79.2% of RA-ILD patients treated with ncs-DMARDs had stable or improved HRCT scans, and the fatality rate due to ILD progression was only 4.9%, providing overall support for the stabilizing effect of these agents, including JAK inhibitors (4).

## Meta-analysis of observational studies

The 2024 meta-analysis by Narváez et al. provides quantitative efficacy data. In a pooled analysis of studies, treatment with JAK inhibitors was associated with a significant increase of 2.07% in percent predicted FVC (pFVC%) and 3.12% in percent predicted DLCO (pDLCO%) (5). Thoracic HRCT scans showed improvement in 11% of patients (5). The pooled proportion of patients experiencing worsening of pre-existing ILD was low, at 5% (5).

## Real-world and recent cohort studies

A 2022 real-world observational study from the Turkish TREASURE registry specifically evaluated tofacitinib in patients with confirmed RA-ILD (17). After a median follow-up of 12 months, the mean percent predicted FVC showed a small but statistically significant improvement (+3.0%, p=0.014), and worsening of FVC was observed in only 11.1% of patients (16). The 2025 MAJIK-SFR national French cohort study prospectively followed 42 RA-ILD patients treated with JAK inhibitors for a median of 21 months (17). The study found no significant changes in FVC or DLCO during follow-up. HRCT lesions remained stable in 69% of patients, improved in 12%, and worsened in 19% (18). This aligns with a 2023 Italian multicenter retrospective study of 43 RA-ILD patients treated with JAK inhibitors, which reported HRCT stability in 86% of patients and FVC stability in 78.6% over a median follow-up of 19.1 months (8). A relevant limitation is the lack of standardized definitions for ILD worsening/improvement across studies (12). Furthermore, different RA-ILD patterns (e.g., UIP vs. NSIP) may respond differently to therapy (12, 19).

## Efficacy in the UIP pattern and comparisons with other therapies

The UIP pattern, associated with a worse prognosis, is the most common in RA-ILD. A 2025 review on immunosuppressive therapy for UIP in autoimmune diseases highlighted that, in contrast to the harmful effects of immunosuppression in idiopathic pulmonary fibrosis (IPF), immunosuppressive treatments (including JAK inhibitors) for RA-associated UIP have shown promising results (19). The MAJIK-SFR study included patients with UIP (43%) and NSIP (46%) patterns, and stability was observed across patterns (18).

In addition to immunosuppressive therapies, antifibrotic agents have emerged as a therapeutic option for progressive fibrosing ILD, including RA-associated UIP. The INBUILD trial demonstrated that nintedanib, a tyrosine kinase inhibitor, reduced the rate of forced vital capacity decline in patients with progressive fibrosing ILD, including those with autoimmune disease-related ILD. Similarly, the TRAIL1 study suggested a potential role for pirfenidone in RA-ILD, though results were less conclusive. Current European guidelines (e.g., EULAR) acknowledge the potential use of nintedanib in selected patients with progressive RA-ILD, particularly those with a UIP pattern unresponsive to immunosuppression. However, direct comparative evidence between JAK inhibitors and antifibrotic agents remains scarce, and their potential synergistic or sequential use warrants further investigation.

Comparative Efficacy with Abatacept: Direct comparative studies are emerging. A 2024 retrospective nested case-control study compared JAK inhibitors (tofacitinib and baricitinib) with abatacept (ABT) in Japanese patients with RA-ILD (20). After propensity score matching, there was no significant difference in drug persistency rates at 24 months (ABT 61.9% vs JAKi 42.8%, p=0.256) or in the incidence of pulmonary complications (p=0.683) between the two groups. Notably, ground-glass opacity (GGO) scores on HRCT significantly improved in the JAK inhibitor group (p=0.03) but not in the ABT group, although the sample size for this analysis was small. Fibrosis scores did not change in either group. Both treatments were equally effective in reducing RA disease activity and enabling glucocorticoid tapering (20). This study suggests that JAK inhibitors have comparable safety and efficacy to abatacept for RA-ILD, with a potential signal for improving inflammatory lung changes (20).

## Safety profile and risk management

The overall safety profile of JAK inhibitors in RA is established, with known risks including infections, venous thromboembolism, and laboratory abnormalities. In RA-ILD patients, who are often older and have compromised lung reserve, safety considerations are paramount (21).

Infection Risk: As highlighted in real-world studies, RA-ILD patients on JAK inhibitors are at increased risk for serious infections, particularly lower respiratory tract infections (12, 17). The MAJIK-SFR study reported 16 episodes (38%) of respiratory tract infections, though most were non-severe (12). The meta-analysis by Narváez et al. reported a pooled adverse event rate of 14% in RA-ILD patients (5). Comprehensively, the literature has not demonstrated a specific, significantly increased risk of pulmonary infection attributable to JAK inhibitors in the context of RA-ILD (12). Vigilance for infection is a general requirement for most immunosuppressive therapies used in RA-ILD, especially in older patients (21).

ILD Events and Acute Exacerbations: A *post-hoc* analysis of tofacitinib trials cited a low incidence rate for ILD events (17). Encouragingly, in the MAJIK-SFR cohort, only one acute exacerbation of ILD was recorded during the 21-month follow-up, and no ILD-related deaths occurred (12) The pooled proportion of patients with worsening pre-existing ILD was 5% (5).

Other Safety Signals: No major cardiovascular events, venous thromboembolism, or lung cancers were diagnosed in the recent cohort studies during their follow-up periods (8, 12). However, sample sizes and follow-up durations may be insufficient to detect these rare events. The known risks from the ORAL Surveillance trial [increased risk of major adverse cardiovascular events (MACE), malignancies, thrombosis] warrant continued caution, especially in patients over 50 with cardiovascular risk factors (22). Older patients with RA-ILD represent a population where these risks require careful individual assessment (21).

## Comparison with TNF inhibitors: mortality and severe outcomes

While comparisons with abatacept show similar safety, a 2025 large-scale, real-world retrospective cohort study using the TriNetX database raised significant concerns when comparing JAK inhibitors to TNF inhibitors (TNFi) specifically in RA-ILD patients (23). After propensity score matching 812 patients in each group, the JAK inhibitor cohort exhibited a significantly higher all-cause mortality risk (HR 1.458, 95% CI: 1.136–1.870). Additionally, the JAK inhibitor group had higher risks of hospitalization (HR 1.167), need for critical care services (HR 1.854), and mechanical ventilation (HR 2.609) (22). Subgroup analyses indicated that the heightened mortality risk was particularly pronounced in patients aged over 65 years (HR 1.815) and in those with pre-existing cardiovascular risk factors (HR 1.636) (23). A sensitivity analysis suggested the increased mortality risk might be driven by early adverse events, but the increased risk of mechanical ventilation remained significant (23) These findings highlight a potential differential safety profile between JAK inhibitors and TNF inhibitors in RA-ILD, especially for older patients and those with cardiovascular comorbidities, suggesting that treatment selection requires careful individualized risk assessment (23). This contrasts with the broader safety profile noted for JAKi in the recent systematic review (1), which found their safety profile favored over csDMARDs/immunosuppressants regarding serious adverse events, though direct comparisons with TNFi were not the focus. This discrepancy underscores the heterogeneity of real-world evidence and the critical importance of comparator choice. For the practicing rheumatologist, this study serves as a critical reminder: while JAK inhibitors may be comparable or favorable versus other non-TNFi biologics, they may carry higher risks than TNF inhibitors in the specific, vulnerable RA-ILD population. This necessitates a nuanced discussion with patients, particularly those >65 or with CV risk factors, where the risk-benefit balance might tilt towards alternative agents like abatacept or rituximab.

## Discussion and concluding remarks future perspectives and conclusion

Current evidence, though evolving, positions JAK inhibitors as a viable therapeutic option for RA patients with concomitant ILD. Their mechanism of action-modulating macrophage polarization to target both inflammation and fibrosis-is mechanistically compelling (11). This is further supported by preclinical evidence showing direct inhibition of the IL-17A/JAK2/NF-κB fibrotic axis in lung fibroblasts (9) and the expansion of immunosuppressive MDSCs in the lung microenvironment (10). The potential of JAK inhibitors in combination therapy, as suggested by the study of tofacitinib plus iguratimod showing improved lung function and HRCT outcomes in RA-UIP, warrants further investigation (13). Clinical data suggest they do not increase the risk of incident ILD (5, 14) and may even be associated with a reduced risk compared to some bDMARDs such as adalimumab (15) and may help stabilize or even improve lung function and radiological findings in established disease (4, 5, 8, 12, 13, 16, 17, 19). Their efficacy and safety appear comparable to other recommended biologic agents like abatacept and rituximab (6, 20).

Another promising yet underexplored avenue is the combination of JAK inhibitors with antifibrotic agents such as nintedanib or pirfenidone. Given the dual pathophysiology of inflammation and fibrosis in RA-ILD, a combined strategy targeting both pathways simultaneously could theoretically offer superior disease control. Preliminary preclinical data suggest that JAK inhibition may modulate macrophage polarization and cytokine profiles, while antifibrotic agents directly inhibit fibroblast proliferation and extracellular matrix deposition. To date, no clinical trials have specifically evaluated such combinations in RA-ILD, representing a significant gap in the current therapeutic landscape. Future studies should explore the safety, tolerability, and efficacy of JAK inhibitor–antifibrotic combinations, particularly in patients with progressive UIP-pattern disease.

Significant gaps remain, as highlighted by the latest systematic review (1): the overall quality of evidence is moderate to low, driven by the predominance of observational studies and inherent biases. Only two RCTs were available for RA-ILD overall, and none specifically designed for JAK inhibitors. Significant heterogeneity was observed across studies, only partially explained by treatment type. No RCTs are specifically designed for RA-ILD and JAKi, though prospective trials (e.g., PULMORA, RAILDTo) are underway (12). Direct comparisons between different JAK inhibitors are lacking, and their optimal place in the treatment sequence is undefined. Furthermore, data on JAK inhibitor combination therapies, such as with iguratimod, are preliminary and require validation in larger, controlled trials (13). Critically, emerging real-world comparative data suggest that JAK inhibitors may be associated with higher risks of mortality and severe respiratory outcomes compared to TNF inhibitors in the RA-ILD population, particularly in those over 65 (23). This underscores the necessity for personalized treatment selection based on patient age, comorbidities (especially cardiovascular), and individual risk profiles, aligning with a patient-centric approach for older adults with RA-ILD (21). The evidence, primarily from observational studies, is considered to have low certainty, underscoring the need for randomized controlled trials (1, 5).

## Conclusion

JAK inhibitors represent a promising oral therapy for RA-ILD with a dual mechanism of action addressing key disease pathways (11). Preclinical studies confirm their potential to block specific pro-fibrotic signaling, such as the IL-17A-driven JAK2/NF-κB pathway in lung fibroblasts (9), and to modulate lung immunity via MDSC expansion (10). Clinical evidence indicates a favorable profile regarding ILD risk (5, 14) and potential for stabilizing existing lung disease, with efficacy comparable to other advanced therapies like abatacept and rituximab (6, 20). Early data on combination therapy with agents like iguratimod suggest additional benefits in improving lung function and imaging in RA-UIP, supporting the concept of dual-targeted therapy (13). Recent prospective and real-world data, including reports of success in refractory cases (16), consistently show that most RA-ILD patients experience stable lung function and HRCT findings on JAK inhibitor therapy, with a low rate of acute exacerbations (8, 12). However, the clinician must weigh these benefits against the established class safety signals. Their use requires meticulous safety management, including vigilance for infections (21) and particularly, careful consideration of the potentially elevated risks of mortality and severe outcomes compared to TNF inhibitors in older RA-ILD patients with comorbidities, as highlighted by recent large-scale comparative data (23). Therefore, treatment decisions should be individualized, incorporating patient age, comorbidities, ILD subtype, and prior treatment history, with ongoing monitoring for both efficacy and safety. Dedicated prospective trials are urgently needed to solidify their efficacy and guide optimal, personalized use in this challenging patient population.
